# A Multi-Scale CNN for Transfer Learning in sEMG-Based Hand Gesture Recognition for Prosthetic Devices

**DOI:** 10.3390/s24227147

**Published:** 2024-11-07

**Authors:** Riccardo Fratti, Niccolò Marini, Manfredo Atzori, Henning Müller, Cesare Tiengo, Franco Bassetto

**Affiliations:** 1 Informatics Institute, University of Applied Sciences Western Switzerland (HES-SO Valais), 3960 Sierre, Switzerland; 2 Department of Neuroscience, University of Padua, 35122 Padua, Italy; 3 Medical Informatics, University of Geneva, 1205 Geneva, Switzerland; 4 The Sense Innovation and Research Center, 1007 Lausanne, Switzerland; 5 Clinic of Plastic Surgery, University Hospital of Padua, 35128 Padova, Italy

**Keywords:** surface electromyography (sEMG), hand gesture recognition, transfer learning, deep learning, domain adaptation, prosthetic hands, real-time systems, user-specific models, gradient-reversal layer, self-supervised learning, NinaPro database, triplet margin loss, fine-tuning, signal processing, adaptive systems

## Abstract

Advancements in neural network approaches have enhanced the effectiveness of surface Electromyography (sEMG)-based hand gesture recognition when measuring muscle activity. However, current deep learning architectures struggle to achieve good generalization and robustness, often demanding significant computational resources. The goal of this paper was to develop a robust model that can quickly adapt to new users using Transfer Learning. We propose a Multi-Scale Convolutional Neural Network (MSCNN), pre-trained with various strategies to improve inter-subject generalization. These strategies include domain adaptation with a gradient-reversal layer and self-supervision using triplet margin loss. We evaluated these approaches on several benchmark datasets, specifically the NinaPro databases. This study also compared two different Transfer Learning frameworks designed for user-dependent fine-tuning. The second Transfer Learning framework achieved a 97% F1 Score across 14 classes with an average of 1.40 epochs, suggesting potential for on-site model retraining in cases of performance degradation over time. The findings highlight the effectiveness of Transfer Learning in creating adaptive, user-specific models for sEMG-based prosthetic hands. Moreover, the study examined the impacts of rectification and window length, with a focus on real-time accessible normalizing techniques, suggesting significant improvements in usability and performance.

## 1. Introduction

Deep learning has achieved important improvements in surface Electromyography (sEMG) recognition systems, supporting the development of sophisticated prosthetic devices, despite the intrinsic complexity of sEMG signals. However, many challenges remain, including catastrophic forgetting, cross-user adaptation, and robustness in real-time performance [1].

Motor neurons play a crucial role in converting brain information into muscle movement [2]. The Motor Unit (MU), which consists of a motor neuron within an innervated muscle fiber, is the fundamental unit of control. The union of numerous MUs generates Motor Unit Action Potentials (MUAPs) [3] that eventually induce movement. The EMG signal acquired from the sensing devices represents the discharge properties of the Motor Units, expressing MUAP convolution in both time and space [4,5]. Typically, EMG signals have an amplitude of around ±5000 μV and a frequency range between 6 and 500 Hz, with the most important frequency power being between 20 and 150 Hz [6]. Surface Electromyography (sEMG) is a non-invasive approach to hand gesture identification in the myoelectric control of prosthetic devices [7].

The key advantage of sEMG over other techniques, such as implanted electrodes, is its simplicity in collecting and analyzing muscle electrical data, allowing users to use their prosthetic limbs naturally through muscle activation patterns that can resemble the user’s original hand movements. Despite substantial developments in this sector, sEMG-based control systems in practical applications still face obstacles [8]. In particular, unpredictable real-life situations that differ significantly from controlled laboratory conditions can bias real-world performance compared to a controlled environment [9,10]. These variations can strongly affect the shape and intensity of the signal, lowering the performance of sEMG-based control systems. Typical examples of these variations include electrode shift [11], unpredictable changes in skin electrode impedance [12], muscle fatigue, and alterations in residual limb posture [13]. As a result, achieving precise control over daily activities becomes challenging.

Many efforts have been made to investigate novel ways to improve the capabilities of myoelectric control, in order to overcome these constraints. Recent advancements have suggested the use of a larger number of sEMG electrodes placed around the residual limb, combined with sophisticated machine learning techniques, [14]. Nevertheless, increasing the number of electrodes complicates the network, increases expense, and may not always be feasible, given the arm’s remaining fraction.

As prosthetic devices improve their functionality to restore the full capabilities of a missing limb, their regulation becomes increasingly challenging [15]. Therefore, new control paradigms and algorithms are being investigated to optimize grasping and manipulation tasks in diverse real-world circumstances.

Deep learning has emerged as a powerful solution in multiple areas of study, such as natural language processing, computer vision, and speech recognition [16]. Deep learning techniques have been applied to improve the precision and reliability of EMG-based gesture recognition systems, and this success has been mimicked in the field of electromyography analysis.

Atzori et al. [17] were among the first to apply Convolutional Neural Networks (CNNs) to recognize EMG patterns. Geng et al. [18] used HD-sEMG data to show the capabilities of CNNs in distinguishing between hand gestures. Their findings revealed how well deep networks can learn complex patterns in this domain. By extending this strategy with a multi-stream divide-and-conquer CNN architecture, Wei et al. [19] increased recognition accuracy over single-stream CNNs.

Hu et al. [20] investigated the incorporation of Recurrent Neural Networks (RNNs) into CNN design, and they presented a hybrid CNN-RNN network. Compared to conventional machine learning methods, this architecture showed improved generalization capabilities using unique sEMG picture representations. Progressive Neural Networks (PNNs) were used by Allard et al. [21] to transfer information from a source domain to a target domain. Based on this, they subsequently improved the PNN technique and demonstrated its efficiency with increasingly complicated gesture datasets [22].

Research has also explored the use of Transfer Learning (TL) to reduce training load and to enhance generalization. By pre-training a source model on various participant data, research has been able to enhance recognition accuracy when applied to the hand motions of a different target participant. Wang et al. [23] specifically proposed an Iterative Self-Training Domain Adaptation (STDA) method for cross-user sEMG recognition, combining discrepancy-based alignment and iterative pseudo-label updates. Islam et al. [24] developed a lightweight All-ConvNet+TL model that efficiently tackles inter-session and inter-subject variability, which successfully enhances the accuracy and speed of sEMG gesture classification, making it well suited for real-time applications. Nguyen et al. [25] took a different approach by introducing a Frequency-based Attention Neural Network (FANN) combined with Subject-Adaptive Transfer Learning.

Despite advances in TL for EMG pattern recognition, several challenges still need to be overcome. Current deep learning solutions have limits, in terms of long-term reliability and adaptability to new gestures or users [26,27]. Indeed, the user can benefit from the option to adapt and/or include new gestures into the model’s repertoire of recognizable gestures. Researchers in this field have shown that deep learning may be the key to enhancing the precision and robustness of sEMG-based control. Problems such as long-term dependability, adaptation to new motions [28], and muscle activation coverage should be addressed as well [29]. Furthermore, in the case of prosthetic devices, user adaptation, energy usage, and inference time must all be considered.

The key objective of this study was to enhance model generalization among patients, to reduce individual variability, hence facilitating quick user adaptation through Transfer Learning. It also targeted key challenges in sEMG-based hand gesture recognition for prosthetics, prioritizing low-computation interfaces and efficient data splitting techniques.

To achieve these goals, different pre-processing steps, including rectification, window length, and normalization procedures, were analyzed. The study also investigated Transfer Learning with few-shot learning for new user fine-tuning. In the end, to evaluate generalization, we employed an adversarial network with gradient-reversal descent [30] and self-supervision with triplet margin loss [31].

## 2. Material and Methods

An extensive analysis of the methodologies adopted is provided in this section. It starts by describing the data used to assess model performance and their acquisition protocol. Two data splitting strategies, inter-subject and intra-subject, are then introduced, followed by a detailed description of the model structure, along with the training strategies employed for pre-training the model backbone. The Transfer Learning framework description is then presented. Finally, data pre-processing, normalization strategies, and parameter tuning methodologies are discussed.

### 2.1. Data Acquisitions

Several publicly accessible datasets were used for the model evaluation, specifically the NinaPro DB2, DB3, and DB7 databases, for which the details are listed in Table 1. The setup used Delsys Trigno electrodes for sEMG data acquisition. The subjects mimicked 40 hand gestures, each repeated six times with 3 s of rest between exercises. Visual stimuli were presented on a laptop, and relabeling was performed to account for delays. The acquisition protocols for these databases are described in further detail in [32].

**Figure 1:**
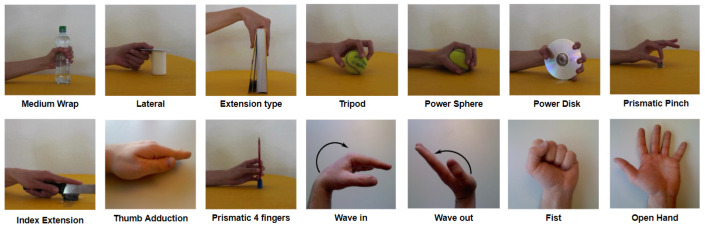
Selected hand gestures from Activities of Daily Living (ADL).

A subset of 14 gestures was selected from the available gestures based on the Activities of Daily Living (ADL) shown in Figure 1. This selection was made to balance the trade-off between number of gestures and accuracy, as the study was tailored for a real prosthetic device.

### 2.2. Data Splitting

The experimental strategy included two data splitting strategies: inter-subject and intra-subject. Inter-subject involved training on data from one group of patients and validating on another; intra-subject involved training, validating, and testing on different repetitions of the same gestures from the same pool of subjects.

For both split modalities, 15 subjects (12 healthy individuals and three amputees) were set aside to be used as test subjects for Transfer Learning. In the first method, to cover a maximum variability, the remaining patients were randomly divided, with 80% used for training and 20% for validation.

With regards to the intra-subject method, three repetitions were used for training (2, 4, 6), two for validation (1, 5), and one for testing (3). This division was introduced to account for variables such as muscular fatigue, which can induce signal fluctuations [33].

Additionally, the 15 subjects initially excluded and selected for testing Transfer Learning could also be used in both splitting methodologies to evaluate model performances over unseen subjects. This enabled a subsequent assessment of the Transfer Learning framework for user-specific fine-tuning. Both the intra-subject and inter-subject approaches applied Transfer Learning to new subjects, allowing a comparative evaluation of their effectiveness.

### 2.3. Data Pre-Processing and Normalization Strategies

The pre-processing involved filtering, windowing, rectification, and normalization. These strategies were selected as they did not require a significant computational effort, which is an important constraint given the expected application for real-time prosthesis use and its associated requirement for energy and efficiency and speed [34]. In order to obtain an informative bandwidth from 10 to 500 Hz, these data were filtered applying a fourth-order bandpass Butterworth filter [35]. Additionally, a notch filter at 50 Hz was applied, to remove power-line interference.

Different windows were employed, each with a 75% overlap. These windows comprised 100, 150, 200, and 250 milliseconds. Using overlapping windows reduced information loss at each window boundary, resulting in a more accurate representation of the signal’s temporal features.

Normalization has long been used to reduce heterogeneity in electromyographic data and to mitigate substantial inter-subject variability [36]. Three normalization strategies (range [0 1], range [−1,1], and Z-Score) were evaluated in two ways: normalization by subject and normalization by subject and channel [37,38].

The first approach relies on the calculation of subject-specific metrics from all channels combined, which includes mean, standard deviation, minimum, and maximum. The second approach, working at the channel level, is less likely to be influenced by artifacts, spikes, and noise-related channels [39].

### 2.4. Model Structure

**Figure 2:**
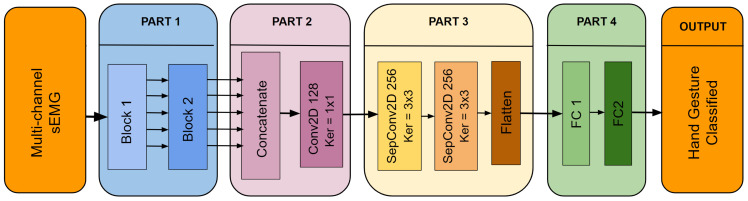
Architecture of the Multi-Scale CNN model.

The architecture of the proposed Multi-Scale Convolutional Neural Network (MSCNN) model is shown in Figure 2. This figure provides a clear representation of the model’s structure and the relationships between its various components. For a more detailed understanding of the data flow within the MSCNN, refer to Table 2, which outlines each step and operation in the process.

The MSCNN model is composed of four distinct parts, each serving a unique purpose. These components are as follows:

**Part 1)**  As seen in Figure 2, this part is divided into two distinct blocks. Block 1 aims to extract features to capture the spatio-temporal patterns in the sEMG signal. The multichannel sEMG signal is represented as an image for each sample.
     A set of five filters, sized (W × 3, 2*W × 3, 3*W × 3, 4*W × 3, and 5*W × 3) are employed in Block 1, where W is selected as 1/20 of the chosen window size. The selection of these sizes was driven by the need to parameterize the model consistently across different window sizes to facilitate parameter tuning and comparison. Larger filter sizes correspond to lower-frequency related characteristics and vice versa.
     Each feature map output from the parallel convolutions of Block 1 is then fed into Block 2. Block 2 has a similar structure and uses five additional convolution layers with fixed 3 × 3 filter sizes. In Block 2, separable convolutions are used to reduce the number of trainable parameters, and the number of neurons in each layer is always doubled compared to the previous ones. Furthermore, the dropout layers and Batch Normalization (BN) layers in both blocks are implemented to increase the MSCNN model’s overall generalizability.
**Part 2)**  The main objective of this part is to reduce the dimensionality of the feature maps that come from Part 1 and then fuse them. After Part 1, the number of feature maps is reduced from 320 (64 × 5) to 128 by using 1 × 1 filters in this convolutional layer. The 1 × 1 convolution prevents the use of fully connected layers, thereby decreasing the number of parameters and preventing overfitting.
**Part 3)**  Part 3 allows the model to extract deeper features by employing other separable convolutional layers, which reduces the number of parameters and, hence, the computational load, an essential requirement for embedded applications.
**Part 4)**  The final stage of the model involves converting the obtained features to class probabilities for classification. This is accomplished by combining two completely connected layers that process the deep features previously collected.

Notably, in *Part 1*, a customized Conv2D layer is used for the first parallel convolutions. This layer employs zero padding along the temporal axis and circular padding along the first 8 channel axes to preserve spatial relationships between electrodes.

The rationale for this approach is evident considering the acquisition setup of the databases, where the initial eight electrodes around the arm are organized in a circular arrangement. This padding accounts for preserving spatial patterns, as converting a circular architecture to a linear representation might result in loss or distortion of vital information.

### 2.5. Model Pre-Training Strategies

This study employed three training strategies for the backbone of the model before applying Transfer Learning: standard training, pre-training with triplet margin loss, and domain adversarial network with gradient-reversal implementation.

Standard training involves a typical classification task using the softmax function and cross-entropy loss at the end of the linear layers, to predict class probabilities.

Triplet margin loss enhances domain adaptation by learning data representations that increase the similarity between positive pairs and separate negative pairs in the embedding space. The loss is defined as

(1) $$L_{\mathrm{triplet}}=\sum_{i}\left[\max\left(0,\mathrm{margin}+d\left(a_{i},p_{i}\right)-d\left(a_{i},n_{i}\right)\right)\right],$$

where $a_{i}$ is the anchor sample, $p_{i}$ is a positive sample, $n_{i}$ is a negative sample, and margin specifies a minimum distance between positive and negative pairings. In our example, a positive sample is considered as a “different subject with the same label”, while a negative sample is simply identified by a different label. This technique drives the model to improve discrimination between subject-specific features, allowing for effective classification in the following trials.

The gradient-reversal technique penalizes the primary model loss function depending on the performance of the domain classifier, encouraging the model to learn features that are domain-invariant (different subjects). The objective function to be reduced is expressed as follows:

(2) $$\min\frac{1}{n}\sum_{i=1}^{n}L_{c}\left(f_{i};y_{i}\right)-\lambda \cdot \frac{1}{m}\sum_{j=1}^{m}L_{d}\left(g_{j};d_{j}\right),$$

Here, $L_{c}$ is the classification loss and $L_{d}$ is the domain classifier loss. The parameter λ determines the trade-off between classification and domain loss. The approach applies an exponential scaling factor to shift the model’s attention from classification task to domain-invariant learning across training epochs.

**Figure 3:**
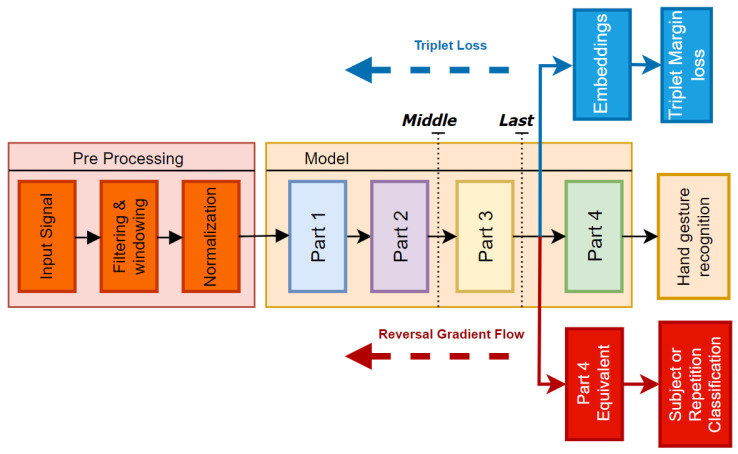
Diagram illustrating the architecture used during pre-training with triplet loss and gradient reversal. The two black dotted lines indicate the portions of the model retrained under two different configurations: *last* and *middle*.

As illustrated in Figure 3, the embeddings for the triplet margin loss are extracted from the output of the flatten layer in *Part 3*, which directly precedes the classification head. In the domain adversarial network with gradient reversal, an additional head, identical to *Part 4*, is integrated into the model architecture connected to the output of *Part 3*. The objective of this secondary head is to classify subjects, thereby encouraging the backbone to become invariant to subject-specific features.

### 2.6. Transfer Learning Framework

Two different fine-tuning approaches were tested, as depicted in Figure 3. In the first approach, referred to as *last*, only the two final linear layers from *Part 4* were retrained, for a total number of ∼70,000 trainable parameters. In contrast, for the second framework, named *middle*, only the *Part 1* and *Part 2* layers were frozen, leading to ∼200,000 trainable parameters.

Experiments were conducted with varying data quantities to be used in retraining (from 1 to 4 repetitions) for each subject. For each amount of data, the model was re-trained five times by varying the combination of repetitions. This allowed us to observe the model’s behavior and its vulnerability to overfitting during the fine-tuning process.

Moreover, dividing data into several repetitions mimicked a real-world online scenario in which the model has no prior on the specific validation and test iterations of the same exercise beforehand. This allowed us to assess the ability to adapt to unseen data more realistically.

To minimize the impact of randomness and to obtain a more reliable comparisons between backbones, a 5-fold cross-validation was used. This means that for each subject in the test group, we trained each pre-trained model five times, each with a different number of repetitions for re-training. In each fold, a different set of repetitions was employed for validation and testing, ensuring the model performance was evaluated on unseen repetition-specific data across all folds.

This approach provided a more robust and reliable assessment of the generalizability of the models and the ability to handle variations in data.

### 2.7. Hyperparameter Optimization Using Grid Search

Hypothetical correlations between normalization techniques, activation function, and rectification were also investigated. Once the model was fully parameterized, three exhaustive grid searches were performed by varying the normalization methods. These searches were used to evaluate the impact and effectiveness of the hyperparameters listed in Table 3. For this experiment, an intermediate window of 300 points, corresponding to 150 ms, was chosen. This ensured that there was sufficient time (≈100–150 ms) to process, infer, and perform movements of the prosthetic hand before reaching the critical threshold of 300 ms, considered as the upper limit for the user to perceive a real-time control [40,41].

Interestingly, one search focused on the effects of the [−1, 1] normalization method, particularly on the impact of forcing values into the positive range (e.g., rectification, Z-score) over information preservation. For more details, see the ‘Hyperparameter Optimization via Grid search’ subsection in the ‘Results’ section.

## 3. Results

This section begins with an evaluation of the effect of the window length. It then presents the results of the grid search technique used for the hyperparameter tuning. Following this, the performance of the model across the databases is showcased, before delving into the main aspect of Transfer Learning.

The *middle* Transfer Learning framework achieved accuracy of 98% ± 0.02 on a new subject after retraining for just two epochs over four repetitions within a 150 ms window, demonstrating the model’s potential for real-time application. The code is available at GitHub.

### 3.1. Window Length Impact

This subsection compares the various window sizes for the intra-subject experiment on the combined database. Windows of size 200, 300, and 400 points, corresponding to 100, 150, and 200 ms, respectively, were tested with the fixed parameters showed in Table 2.

Furthermore, a 20 ms window (comprising 40 points) was chosen, considering the potential use of a majority voting strategy, as this has been shown to be a valid option to improve reliability [42]. The results shown in Table 4 highlight the significant performance drops associated with the smallest window size, as expected. No substantial differences were observed among the other three window sizes. Consequently, subsequent experiments were conducted using only the 200- and 300-point windows, as they are more suitable for real-time prosthesis control.

### 3.2. Hyperparameter Optimization via Grid Search

From the very first experiments, the channel-dependent normalization mode revealed a clear benefit, since it was better at mitigating errors or abnormalities that were present in some channels. For this reason, the results presented were all performed with channel-wise normalization.

The grid search was performed employing several normalization techniques. For this evaluation, the standard training algorithm was used over the merged database with intra-subject splitting.

Table 5 summarizes the best three configurations identified using three grid searches with changing normalization techniques. For each of them, 20 models were generated, employing a fixed window length of 300 time-points, that is, with a 2000 Hz Delsys Trigno Device, 150 ms per window. For better readability, the table excludes the parameters that seemed to have minimal impact on performance. In particular, parameters such as the number of neurons in N_Multi-kernel, N_Post-Concatenation, and the maxpool type were omitted, as they were consistent across the top three configurations.

Compared to other normalization methods, Z-score normalization exhibited better accuracy and faster convergence, in line with other studies [43]. However, the top three configurations when exploiting normalization within the range (−1; 1) and no rectification achieved similar performance with half the number of neurons in the *N_Separable_Conv* layers of *Part 3* of the architecture. This suggests that accounting for negative values may allow the model to learn effectively with a simpler architecture. In contrast, the last three rows show that rectifying the value and then normalizing between (−1; 1) might distort information, leading to important performance drops.

### 3.3. Model Performance Across Databases

In Table 6, the performance of the fine-tuned standard model with respect to the different databases for the intra-subject modality are summarized.

DB3 exhibited the lowest performance, likely due to higher variability among amputee subjects.

Moreover, there was a noticeable shift in the dynamics of the performance, especially for DB2 and DB7. Interestingly, DB7 had the best precision, despite having two amputee patients. One possible explanation for this phenomenon could be that because DB7 had only 22 subjects, compared to the 40 of DB2, the model tended to overfit on the subjects employed, which improved its overall accuracy.

Meanwhile, because DB3 was included in the composite database, its performance inevitably suffered as a result. Still, a respectable level of accuracy was obtained, most likely as a result of exploiting more data during training, which typically results in the development of more reliable models. To leverage the benefits of a more comprehensive data pool, all future experiments will be performed using the merged database.

### 3.4. Transfer Learning Framework Effectiveness

In this section, we examine the effects of several techniques on final-user fine-tuning within the Transfer Learning framework. In this context, final-user fine-tuning refers to the process of personalizing the model for users who will be using a myoelectric-controlled prosthesis for hand gesture recognition. The previously excluded subjects are used as new, unseen patients for applying Transfer Learning and re-training on the subject-specific data. All the training was conducted using a Tesla V100-PCIE-32GB GPU (NVIDIA Corporation, Santa Clara, CA, USA) with CUDA version 12.1. The average inference time, measured over 100 single-batched samples, was 3.16 ± 0.31 milliseconds.

Table 7 shows the *last* experiment, where only *Part 4* of the model architecture was retrained. The results show the average and standard deviation over the 15 subjects that were previously discarded from training and validation. The F1 Score is also shown, to account for the slightly imbalanced dataset. This table includes several window sizes, pre-training algorithms, and splitting modalities.

The table provides useful insights. First, the intra-subject partitioning produced the best results for regular training. This was most likely due to the model’s ability to train over multiple epochs, as opposed to patient splitting, which stops early due to overfitting over training subjects.

The inter-subject splitting precluded the model from learning invariant features during standard training. In this scenario, the adversarial network outperformed all the others, showing that it had successfully learned some invariant properties. This pattern was also seen in the reverse amputee training, which ranked second in the inter-subject trials.

Self-supervision with triplet margin loss performed poorly. During pre-training, it did not use task loss, and simply forcing embeddings with triplet loss did not provide useful insights into this challenging problem.

**Figure 4:**
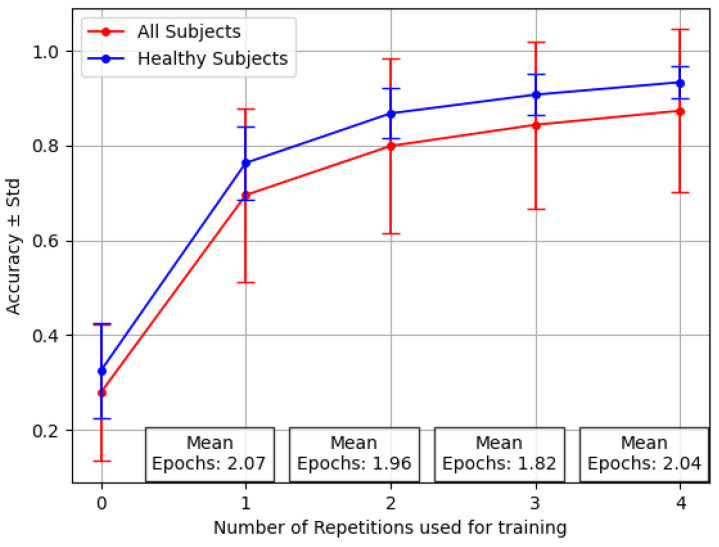
Performance averaged across 12 healthy patients and three amputees for the Transfer Learning *last* with varying numbers of repetitions. Where X=0 (no retraining), this reflects the average results of testing over new, unseen subjects.

Figure 4 shows the variability among individuals, with a focus on the stronger ones between amputees. This graph, based on the inter-subject experiment with the reversal backbone, shows the difference in accuracy values for healthy people alone versus the complete group, including amputees.

The increased standard deviation observed when the pool of 15 subjects included the 3 amputees is clear, mostly considering that the number of amputees was 3 out of 15. In particular, the number of epochs averaged across all the individuals was, on average, less than two, demonstrating that Transfer Learning does not require extensive and computationally expensive training sessions. However, the success of Transfer Learning relies on the consistency of the data used during pre-training within the selected subject.

**Figure 5:**
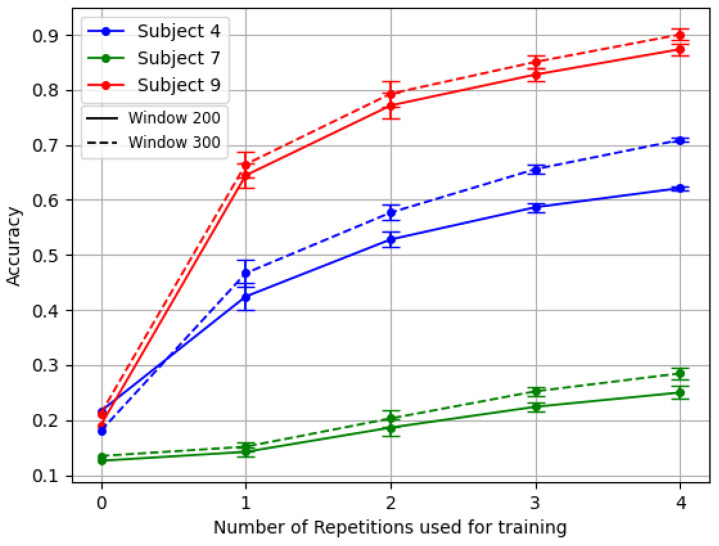
Comparison of the TL *last* for the three amputees. Model backbone pre-trained with reversal gradient and inter-subject splitting technique.

Figure 5 exemplifies this challenge. It showcases the results of the TL framework for the three amputees with the reversal backbone pre-trained with inter-subject splitting.

Table 8 shows the *middle* experiment, where both *Part 3* and *Part 4* of the model’s architecture were re-trained. The results show the average and standard deviation over the 15 subjects that were previously discarded from training and validation. The F1 Score is also shown, to account for the slightly imbalanced dataset. This table includes several window sizes, pre-training algorithms, and splitting approaches.

**Figure 6:**
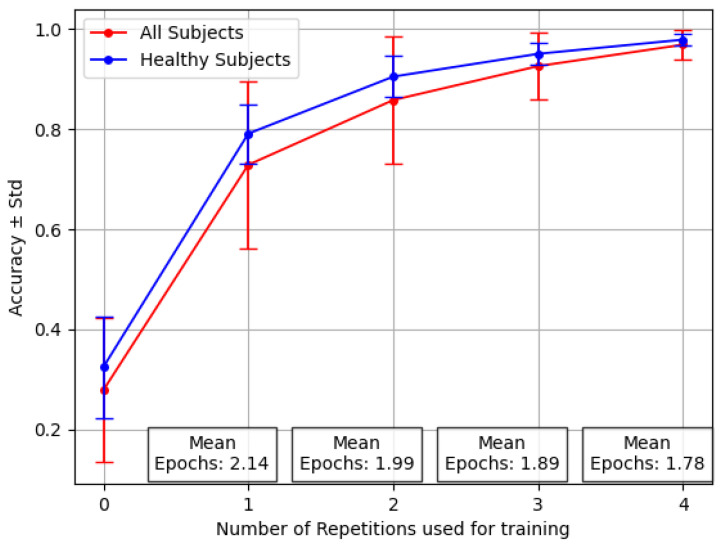
Performance averaged across 12 healthy patients and three amputees for the Transfer Learning *middle* with varying numbers of repetitions.

**Figure 7:**
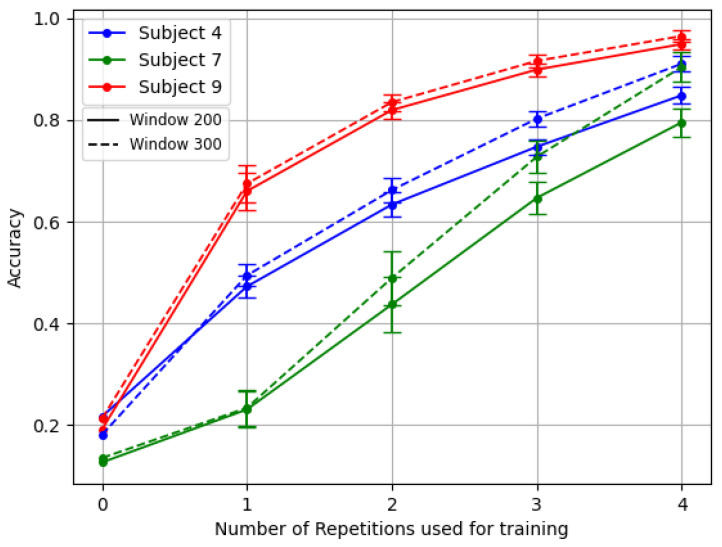
Comparison of the TL *middle* for the three amputees. The Model backbone was pre-trained with gradient reversal and inter-subject splitting.

In Figure 6, it is possible to observe the difference in both mean and standard deviation for accuracy and F1 Score, with respect to Figure 4. Similarly, in Figure 7 it is possible to appreciate such a consistent change in performance even for the three amputees, in particular for the problematic subject number 7.

Table 9 compares our results with other studies on subject-specific Transfer Learning. Direct comparisons are challenging, due to the differences in chosen classes, input data formats, model architectures, number of parameters, and Transfer Learning techniques. However, this table provides our findings alongside recent work in the field. It aims to place our results in the broader context of subject-specific Transfer Learning research.

## 4. Discussion

The results indicate that a more personalized model that learns subject-specific features rather than merely retraining the final linear layers is crucial for improving the adaptability and performance of sEMG-based prosthetic hands. Nonetheless, even in this situation, the number of epochs before overfitting were, on average, fewer than two in all cases, suggesting the potential for efficient and embedded fine-tuning. Given the approximately ≈3 ms inference time, even on a powerful GPU that is not intended for embedded devices, this performance suggests that the system is sufficiently fast for real-time applications or even for a major voting strategy.

Another consideration regards the amputees. These patients present unique challenges for gesture recognition, due to greater differences in their physiological signals, resulting from factors such as the percentage of muscle remaining after amputation along with residual sensations of the phantom limb [51]. Therefore, the creation of user-adaptive models is a crucial area of research in the field of EMG-based prosthetic devices.

For example, subjects number 7 and number 4 of DB3 demonstrated this variability particularly well, as shown in Figure 5. Subject 7 exhibited a complete absence of phantom limb sensation and zero percentage of remaining forearm, leading to the usage of 10 channels instead of 12. Meanwhile, patient number 4 had a unique combination of low phantom sensation, no prior myoelectric experience, and the highest DASH score in the dataset. In contrast, patient number 9 represented better the average clinical profile of the other amputee subjects.

Factors like phantom limb sensation intensity, remaining forearm percentage, DASH score, and prior myoelectric prosthesis experience seemed to influence the model’s ability to adapt to new users.

## 5. Conclusions

This study demonstrates that Transfer Learning techniques improve the effectiveness of sEMG-based prosthetic hands by customizing models to individual users. Retraining with more layers produces better outcomes, emphasizing the benefit of allowing models to learn subject-specific features. The findings underline the importance of managing intra-subject heterogeneity, particularly among amputees, in order to maintain consistent performance between individuals.

Strategies such as adversarial network with gradient-reversal descent and channel-wise normalization can help in building a more robust pre-trained model. Furthermore, allowing an embedded device to extract daily parameters for normalization and re-training in loco should also be considered. Implementing these ideas in prosthetic devices could result in more adaptive, efficient, and user-specific solutions, thereby improving the functionality and usability of these technologies.

### Future Work

During the re-training phase in both modalities, we used standard training, and we split the data by repetitions. However, to account for intra-subject variability, it is possible to retrain layers using gradient-reversal techniques across different repetitions or sessions of the same subject.

This approach would help to maintaining performance consistency and adapt to the user’s unique patterns over time.

Furthermore, in order to overcome performance degradation due to daily variations in prosthetic devices, built-in functions should be implemented. These implementation tools could either make it easier to acquire new data for fine-tuning or provide alternative techniques to avoid such computation on the embedded device itself.

In our case, the alternative would be to collect a single sample for each exercise, which could then be used to quickly update the necessary values for normalizing each channel, based on daily circumstances. Using this single-sample strategy, the system might adjust to daily variations without incurring computational overhead, resulting in efficient and effective performance in real-world scenarios.

In the future, fuzzy similarity formulations [52] could improve sEMG pattern discrimination, whilst adaptive fuzzification techniques [53] could improve system adaptability. These advances have the potential to improve prosthesis control, particularly for smooth multi-gesture recognition.

Improving inference time is a major concern for future work, with a focus on faster computations and reduced energy consumption for prosthetic devices. Faster inference time would also enable the implementation of major voting strategies, which could significantly increase the system’s reliability. Promising advances have been made in this regard by establishing quantization techniques, which may slightly reduce accuracy but dramatically improve energy efficiency and inference time in prosthetic devices [54,55].

## Figures and Tables

**Table 1:** Description of NinaPro databases used. The subjects column refers to the number of patients performing the gestures, while repetitions refers to the number of iterations of the same gesture.

Name	Subjects	Channels	Classes	Repetitions	Frequency	Device
DB2	40	12	41	6	2000 Hz	Delsys Trygno
DB3	11 (A)	12	41	6	2000 Hz	Delsys Trygno
DB7	20 + 2 (A)	12	41	6	2000 Hz	Delsys Trygno

Note: In the context of this study, the letter A in *Subjects* designates amputees.

**Table 2:** Detailed structure of the model and data flow. The number before the layer type represents the number of parallel layers of the corresponding type, e.g., 5-Conv2D represents five conv2D layers. The first dimension (kernel size) of the first set of parallel Conv2D_Circular8 layers increases linearly from a lower value to an upper value. The increment between each kernel size is W = window length/20 (window length fixed at 300 here).

Part	Layer Type	Kernel Size	Output Size	Activ	Options
	1-Input	-	1/300 × 12	-	-
	5-Conv2D_Circular8	[15–75] × 3	32/300 × 14	ReLU	pad = ‘circular’
	5-BatchNorm2D	-	32/300 × 14	-	-
	5-MaxPooling2D	15 × 1	32/20 × 14	-	-
1	5-Dropout2D	-	-	20 × 14	rat e = 0.2
	5-SeparableConv2D	3 × 3	64/20 × 14	ReLU	pad = 0
	5-BatchNorm2D	-	64/20 × 14	-	-
	5-MaxPooling2D	2 × 2	64/10 × 7	-	-
	5-Dropout2D	-	64/10 × 7	-	rate = 0.2
2	1-Concatenate	-	320/10 × 7	-	-
	1-Conv2D	128/1 × 1	128/10 × 7	ReLU	-
	1-SeparableConv2D	3 × 3	256/10 × 7	ReLU	pad = 0
	1-BatchNorm2D	-	256/10 × 7	-	-
	1-MaxPool2D	2 × 2	256/5 × 3		-
3	1-Dropout2D	-	256/5 × 3	-	rate = 0.2
	1-SeparableConv2D	3 × 3	256/5 × 3	ReLU	pad = 0
	1-BatchNorm2D	-	256/5 × 3	-	-
	1-MaxPool2D	2 × 2	256/2 × 1	-	-
	1-Dropout2D	-	256/2 × 1	-	rate = 0.2
	1-Flatten	-	512	-	-
4	1-Linear	-	128	ReLU	-
	1-Linear	-	14	SoftMax	-

**Table 3:** This table presents the hyperparameters explored in the grid search. The keys *N_Multi-kernel*, *N_Post-Concatenation*, and *N_Separable_Conv* represent the number of neurons in the hidden convolutional layers of *Parts 1*, *2*, and *3*, respectively. Notably, in *Part 1*, Block 2 always employed twice as many hidden neurons as Block 1. The **bold** values are the ones selected for the tuned model.

Hyperparameter	Values Chosen
Batch size	64, 128, **256**, 512
Optimizer	SGD, **Adam**
Learning rate	$1\times 10^{-2}$, $1\times 10^{-3}$, $1\times 10^{-4}$, $1\times 10^{-5}$
Decay rate	0, $1\times 10^{-2}$, $1\times 10^{-3}$, $1\times 10^{-4}$, $1\times 10^{-5}$
Activation function	ReLu, **LeakyReLU**, ELU, PReLU
Pooling type	**MaxPooling**, AveragePooling
N_Multi-kernel	16, 32, **64**, 128
N_Post-Concatenation	64, **128**, 256
N_Separable_Conv	64, 128, **256**

**Table 4:** Performance metrics for several window lengths over the merged database. Bold values indicate the best in each metric.

Window Length (ms)	Validation Loss	Accuracy	F1 Score	Best Epoch
100	0.976 ± 0.011	0.775 ± 0.012	**0.720 ± 0.010**	62 ± 4.2
150	**0.971 ± 0.017**	**0.778 ± 0.015**	0.713 ± 0.012	51 ± 3.7
200	0.988 ± 0.019	0.769 ± 0.016	0.718 ± 0.014	56 ± 3.5
20	1.340 ± 0.025	0.689 ± 0.024	0.607 ± 0.019	36 ± 4.7

**Table 5:** The three best model configurations resulting from grid searching, varying the normalization strategies.

Norm Type	Rectification	Accuracy	F1 Score	N_SepConv	Activation
Z_score	Yes	0.8068	0.7514	256	PReLU
Z_score	Yes	0.7971	0.7451	256	LeakyReLU
Z_score	Yes	0.7893	0.7325	256	LeakyReLU
Range (−1; 1)	No	0.7826	0.7280	128	ELU
Range (−1; 1)	No	0.7722	0.7217	128	ELU
Range (−1; 1)	No	0.7758	0.7187	256	LeakyReLU
Range (−1; 1)	Yes	0.7399	0.6692	128	PReLU
Range (−1; 1)	Yes	0.7487	0.6749	128	PReLU
Range (−1; 1)	Yes	0.7338	0.6610	128	PReLU

**Table 6:** Performance metrics over different databases.

Database	Window 100 ms		Window 150 ms	
	Accuracy	F1 Score	Accuracy	F1 Score
DB3	0.503 ± 0.012	0.454 ± 0.004	0.508 ± 0.022	0.458 ± 0.012
DB2	0.824 ± 0.010	0.770 ± 0.016	0.819 ± 0.009	0.768 ± 0.016
DB7	0.856 ± 0.005	0.797 ± 0.004	0.862 ± 0.007	0.796 ± 0.010
Merged	0.789 ± 0.011	0.741 ± 0.009	0.798 ± 0.014	0.768 ± 0.011

**Table 7:** Average performance for the 15 previously excluded patients for TL-*last* experiments, by retraining only *Part 4* for four repetitions. Results are shown for the two pre-training data splitting approaches: inter-subject and intra-subject. Bold values indicate the best performance across different configurations.

Backbone Training	intra-Subject				Inter-Subject			
	Window 100 ms		Window 150 ms		Window 100 ms		Window 150 ms	
	Accuracy	F1 Score	Accuracy	F1 Score	Accuracy	F1 Score	Accuracy	F1 Score
Standard	**0.87 ± 0.20**	**0.86 ± 0.20**	**0.90 ± 0.18**	**0.90 ± 0.18**	0.83 ± 0.19	0.83 ± 0.19	0.86 ± 0.19	0.86 ± 0.19
Pre-triplet	0.66 ± 0.19	0.66 ± 0.20	0.68 ± 0.19	0.67 ± 0.20	0.53 ± 0.19	0.50 ± 0.20	0.66 ± 0.18	0.65 ± 0.19
Reversal	0.84 ± 0.18	0.84 ± 0.19	0.87 ± 0.17	0.87 ± 0.17	**0.85 ± 0.18**	**0.84 ± 0.18**	**0.88 ± 0.16**	**0.87 ± 0.16**
Reversal Amputee	0.84 ± 0.19	0.84 ± 0.20	0.88 ± 0.18	0.87 ± 0.18	0.84 ± 0.19	0.83 ± 0.20	0.87 ± 0.18	0.87 ± 0.19

**Table 8:** Average performance for the 15 previously excluded patients for the TL-*middle* experiments, by retraining both *Part 3* and *Part 4* for four repetitions. Results are shown for the two pre-training data splitting approaches: inter-subject and intra-subject. Bold values indicate the best performance across different configurations.

Backbone Training	Intra-Subject				Inter-Subject			
	Window 100 ms		Window 150 ms		Window 100 ms		Window 150 ms	
	Accuracy	F1 Score	Accuracy	F1 Score	Accuracy	F1 Score	Accuracy	F1 Score
Standard	**0.98 ± 0.02**	**0.98 ± 0.02**	**0.98 ± 0.02**	**0.98 ± 0.02**	**0.95 ± 0.04**	**0.95 ± 0.04**	0.96 ± 0.03	0.96 ± 0.03
Pre-triplet	0.94 ± 0.04	0.94 ± 0.04	0.95 ± 0.03	0.95 ± 0.03	0.93 ± 0.05	0.93 ± 0.04	0.95 ± 0.02	0.95 ± 0.02
Reversal	0.95 ± 0.05	0.95 ± 0.05	0.97 ± 0.03	0.97 ± 0.03	0.95 ± 0.05	0.95 ± 0.06	**0.97 ± 0.03**	**0.97 ± 0.03**
Reversal Amputee	0.95 ± 0.05	0.95 ± 0.05	0.97 ± 0.03	0.97 ± 0.03	0.94 ± 0.05	0.94 ± 0.05	0.96 ± 0.03	0.96 ± 0.03

**Table 9:** Performance comparison of various Transfer Learning frameworks using NinaPro databases.

Citation	Database	Classes	Input	Window	Model	Accuracy %
Zhai 2017 [44] ^1^	DB2	10	Spectrogram	200 ms	2D-CNN	90.06
	DB3					85.53
Wei 2019 [19] ^2^	DB2	50	Features	200 ms	Multiview 2D-CNN	83.7
Soroushmojdehi 2022 [45] ^3^	DB2	17	Raw sEMG	250 ms	PFCnet	82.87
Lehmler 2022 [46] ^4^	DB2	17	Raw sEMG	200 ms	1D-CNN	68.52
	DB3					50.72
Zabihi 2023 [47] ^4^	DB2	17	Raw sEMG	200 ms	TraHGR	88.72
Qamar 2024 [48] ^5^	DB2	50	Spectrogram	200 ms	DP-CNN	94.00
	DB3					85.36
Fan 2023 [49] ^6^	DB2+TL DB3	17	Feature	100 ms	CNN	67.5
Lin 2023 [50] ^6^	DB2+TL DB3	17	Features	200 ms	LE-CNN	83.5
Ours	DB2+DB3+DB7	14	Raw sEMG	150 ms	MSCCNN-last	98.04
					MSCNN-middle	87.70

Overview of Transfer Learning strategies implemented: ^1^ Adaptive self-calibration system utilizing a consensus-based voting mechanism across four separate repetitions. ^2^ Personalized adaptive Batch Normalization applied to each subject’s data. ^3^ Merging and freezing subject-specific and multi-subject features previously extracted before employing a new classifier training phase. ^4^ Retaining the weights of earlier layers while retraining the final layers of the model. ^5^ Subject-specific training from scratch without pre-existing models. ^6^ Initial training performed on DB2, with subsequent layer freezing and retraining of the final layers using DB3 subject-specific data.

## Data Availability

NinaPro data are available at: http://ninaweb.hevs.ch/.
